# Balance Training with Weight Shift-Triggered Electrical Stimulation for Stroke Patients: A Randomized Controlled Trial

**DOI:** 10.3390/brainsci13020225

**Published:** 2023-01-29

**Authors:** Kyeongjin Lee

**Affiliations:** Department of Physical Therapy, College of Health Science, Kyungdong University, Wonju 24764, Republic of Korea; kjlee@kduniv.ac.kr

**Keywords:** stroke, electrical stimulation, weight-bearing, balance

## Abstract

This study aimed to determine the effects of balance training with weight shift-triggered electrical stimulation to improve balance, lower-extremity motor function, and activities of daily living in patients with stroke. The participants were randomly allocated to the balance training with electrical stimulation group (BT-ESG, *n* = 29) or the balance training group (BTG, *n* = 30). Both groups were trained 5 times per week for 6 weeks for 50 min per session. To evaluate static balance, postural sway was assessed and dynamic balance was assessed using the Berg Balance Scale (BBS), Timed Up and Go (TUG) test, and functional reach test (FRT). Lower-extremity motor function was assessed using the Fugl–Meyer assessment. Daily activities were assessed using the Modified Barthel Index. As for static balance, BT-ESG showed a significant improvement compared to BTG in postural swat in both the eyes-open (velocity moment; effect size, 0.88; 95% confidence interval, −1.16 to −1.30), or eyes-closed state (velocity moment; effect size, 0.81; 95% confidence interval, −1.22 to −0.27). Dynamic balance, which includes TUG (effect size, 0.90; 95% confidence interval, −4.67 to −1.25), BBS (effect size, 1.26; 95% confidence interval, −2.84 to 6.83), and FRT (effect size, 1.45; 95% confidence interval, 1.92 to 4.08), in addition to lower-extremity motor function (effect size, 1.38; 95% confidence interval, 2.25 to 4.97), and activities of daily living (effect size, 2.04; 95% confidence interval, 2.04 to 937), showed significant improvement in BT-ESG compared to BTG. These results suggest that balance training with weight shift-triggered electrical stimulation effectively improves balance, lower-extremity motor function, and activities of daily living in patients with stroke.

## 1. Introduction

Postural symmetry is essential for motor system function. The ability to transfer weight from one leg to the other is a fundamental component of walking and activities of daily living (ADL) [1]. Significant problems in chronic stroke patients include asymmetrical weight-bearing, body imbalance, and defects in weight transfer ability [2]. Patients with hemiplegia due to stroke show unbalanced weight-bearing, with less than 25–43% of the body weight on paretic limbs in the standing position [3,4,5]. Chronic stroke patients adopt compensatory strategies and an asymmetrical posture that puts less weight on the paretic limb, thereby not putting weight on the paretic limb [2,6]. This imbalance between the paretic and non-paretic limbs significantly reduces the walking ability of chronic hemiplegic patients, putting them at risk of falls and limiting their ADL [6,7]. The ideal goal in the functional rehabilitation of stroke patients is to restore their walking ability by maintaining a symmetrical posture with even weight support [2].

Electrical stimulation has been used for various purposes in the rehabilitation of stroke patients. It is an effective method for improving balance and walking ability and restoring posture and motor function [8]. Neuromuscular electrical stimulation induces muscle contraction on the paralyzed side and is widely used to prevent foot drop during gait training in patients with stroke [9]. Electrical stimulation induces functionally useful movements in muscles that cannot control voluntary movements in the central nervous system, thereby increasing the strength of specific muscles [10].

Electrical stimulation reportedly has the ability to improve the lower-extremity motor function of stroke patients [9,10]. However, it is necessary to induce muscle contraction at a specific time because equal weight-bearing is delayed, but current methods are insufficient. Thus, an electrical stimulation intervention method that can improve weight distribution and postural control ability between the paretic and non-paretic sides of stroke patients is needed.

An intelligent healthcare system that can help improve health based on IT technology has recently emerged [11,12]. In particular, as smartphones have become popular, technology that connects wearable devices that record health-related information and smartphones to provide this information has developed [13]. Wearable devices help health management by collecting data on patterns such as the number of steps, amount of physical activity, movement, and posture [14]. The use of wearable devices in the rehabilitation area can effectively promote motor learning by providing information accurately and quickly in real time [14].

Previous studies demonstrated the effectiveness of weight transfer training through biofeedback in hemiplegic patients at improving balance and walking ability [15]. As such, the augmentative feedback-based training method, which is useful in clinical practice, is being studied in various ways to improve patient function [16]. Stroke patients wore smart shoes and a wearable device and were trained with reinforcement feedback to reduce weight dependence of the non-paretic limb and improve gait asymmetry in the increased stance and single-support phases of the paretic limb [17,18].

Therefore, we hypothesized that weight dependence focused on the non-paretic limb could be controlled using balance training that induces equal weight support with electrical stimulation feedback, along with a pneumatic-pressure insole. We also believed that it would improve balance, lower-extremity motor function recovery, and ADL in patients with stroke. This study aimed to investigate the effects of balance training with weight shift-triggered electrical stimulation on static balance, dynamic balance, lower-extremity motor function recovery, and ADL in hemiplegic patients.

## 2. Materials and Methods

### 2.1. Subjects

Participants were recruited from chronic stroke patients hospitalized at S Hospital in Seoul, South Korea by publicizing the research purpose and inclusion criteria. In addition, the current study recruited late chronic phase patients whose onsets were more than a year without spontaneous recovery to observe the effects of intervention even more clearly. Among stroke patients, those who understood verbal instructions and had a Mini-Mental State Exam score of ≥24 were included. Those who could stand independently without assistance, with a Brunnstrom motor recovery level ≥ 4, with severe neglect or musculoskeletal abnormalities, with superficial metal (e.g., staples, pins and external fixators), who were suffering from cerebellar disease or dizziness, or with cardiovascular disease were excluded.

Among the patients who wished to participate, those who met the selection criteria were given an explanation of the study purpose, procedures, and precautions and were included upon completing a voluntary consent form. The study was approved by the Research Ethics Committee of Kyungdong University.

This was a randomized controlled trial. Because the intervention was an exercise, blinding of the therapist was not performed, and only the assessor was blinded so that the subject did not know the group allocations. A pilot study using the same exercise protocol was conducted to determine the sample size. Outcome measures in the pilot study were the Timed Up and Go (TUG) test, Berg Balance Scale (BBS), and functional reach test (FRT). We determined the effect size (f = 0.29) based on the value of the TUG test from the pilot study. The alpha error was set to 0.05, and the power was set to 0.8. Therefore, a total of 26 participants were required. Considering a dropout rate of 10%, 30 participants per group were selected. G* Power version 3.19 (Heinrich Heine University Düsseldor, Düsseldorf, Germany) was used for the sample size calculations.

### 2.2. Experimental Procedure

Seventy-five patients were recruited for the study. After receiving an explanation of the study procedure, eight people refused to participate, while another seven were excluded because they did not meet the selection criteria. Sixty subjects were randomly assigned to the balance training with electrical stimulation group (BT-ESG; *n* = 30) or the balance training group (BTG; *n* = 30). To minimize selection bias, a computer program called Random allocation software (version 2.0) (M. Saghei, Isfahan, Iran) was used [19], and the ratio of the two groups was 1:1, and the sequence of patients was in the order of patient number.

Subjects in the BT-ESG performed balance training for 50 min 5 times per week for 6 weeks using a weight shift-triggered electrical stimulation device. Subjects in the BTG performed balance training for 50 min 5 times per week for 6 weeks without a weight shift-triggered electrical stimulation device. Pre- and post-tests were conducted to evaluate the effectiveness of training before and after intervention. The primary outcome was static balance and dynamic balance, and lower-extremity motor function and ADL were evaluated as a secondary outcome. All evaluations were performed in triplicate and averaged by three physiotherapists. All tests were performed by raters blinded to the participants’ information. Participants who were unable to continue the program due to changes in their medical condition during the intervention period and those participating in less than 80% of the total program were excluded from the final study. In the BT-ESG, one patient who was transferred to another hospital during the experiment was excluded, while no subjects in the BTG dropped out.

**Figure 1:**
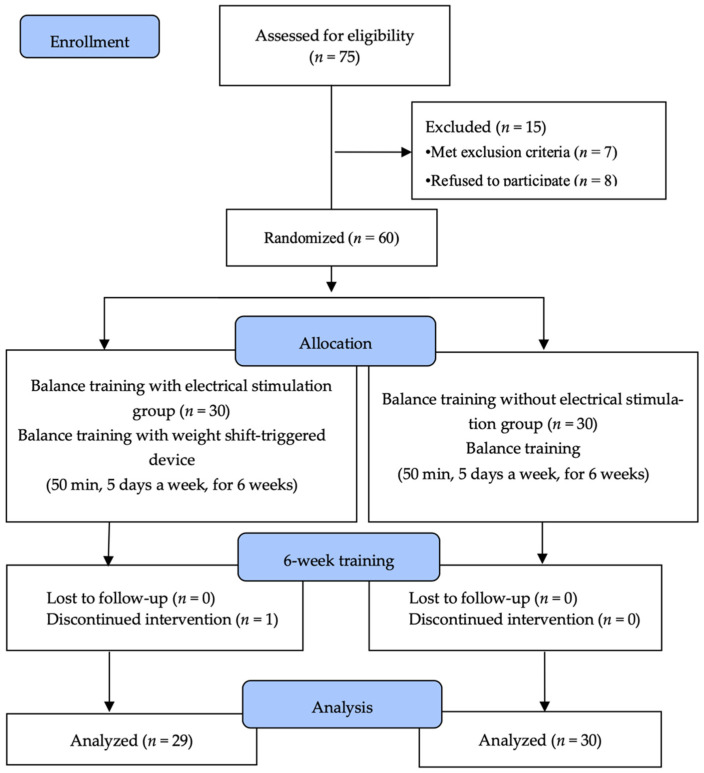
Flow diagram of the study.

Finally, 29 individuals from the BT-ESG and 30 from the BTG participated. All subjects underwent pre- and post-tests, and data from these tests were statistically analyzed (Figure 1).

### 2.3. Experimental Method

#### 2.3.1. Weight Shift-Triggered Electrical Stimulation Device

The weight shift-triggered electrical stimulation device triggers a low-frequency output and induces muscle contraction when a decrease in the pressure signal is detected by the insole pressure-measuring device inside the shoe.

We developed an insole-type pressure-measuring device to measure the weight shift in stroke patients. The device measured the air pressure recorded by the weights of both feet. The weight-bearing ratio is calculated by converting the force applied to the affected and unaffected sides from the sum of both feet. The device consisted of an insole with an air tube, a central device, and a control unit. The air insole measures the pressure caused by the air cap inserted into the sole using an air pressure-measuring device. It wirelessly transmits signals to the central device, which sends signals from both feet to the control device. The control device was developed as a smart phone application.

**Figure 2:**
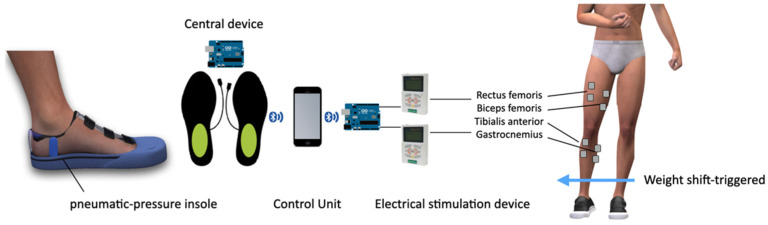
Weight shift-triggered electrical stimulation device.

When the patient tried to transfer the weight from the healthy side to the affected side, the weight support rate of the healthy side decreased, and the electric stimulation device operated as a trigger. Two electrical stimulation devices (EMS-1000; Cyber Medic, Iksan, Republic of Korea) with two channels were used. Electrodes were attached to the rectus femoris, biceps femoris, gastrocnemius lateral head, and tibialis anterior muscle bellies, which are important muscles for weight bearing [20,21,22]. Activation was checked every time training was performed, and the position of the electrodes was attached. All electrodes were square hydrogel electrodes (HRTS50AP 50 × 54 mm^2^; Hurev, Wonju, Republic of Korea). When 90% of the measured value was reached in the insole-type measuring device on the unaffected side, the electrical stimulation device was triggered and the threshold value was adjusted according to individual characteristics. Electrical stimulation was delivered to the muscles through electrodes when the input stimulation reached a threshold. A rectangular two-phase pulse with a pulse width of 300 μs was used. Pulse intensity (mA) was chosen to elicit contraction of the affected limb according to the target intensity (5–60 mA; mean, 16–18 mA). The standard current frequency is 30–35 Hz. Patients were asked to contract lower-extremity muscles when actively supporting weight. The intensity of the electrical stimulation was below the level of inducing muscle contraction. The intensity was adjusted for each patient according to the patient’s muscle contraction ability and sensory state (Figure 2).

#### 2.3.2. Balance Training Program

The balance training program was designed to improve weight shift in stroke patients and was based on the exercise programs of previous studies. The total exercise time was 50 min and consisted of 10 min of warm-up, consisting of stretching and a range of motion exercises to relieve spasticity and leg muscle massage, 30 min of main exercise with weight shift as the main exercise, and 10 min of cool-down. The weight shift exercises included the following: (1) sideways weight shift; (2) diagonal forward shift; (3) diagonal back weight shift; (4) weight shift up a short flight of stairs; (5) weight shift down a short flight of stairs; and (6) sideways weight shift on the platform. Each patient’s functional ability was evaluated and the program was configured accordingly.

### 2.4. Outcome Measurements

#### 2.4.1. Static Balance Ability

A postural assessment system (GB300; Metitur Ltd., Jyvaskyla, Finland) was used to measure static balance ability. The system consisted of a movable triangular platform for feet marked with a ruler on the platform for proper foot positioning. It was used to determine the balance and rehabilitation outcomes and administer the training. This instrument has been widely used to measure balance in adults, the elderly, and stroke patients [23,24,25]. In the test–retest method, the intraclass correlation coefficient (ICC; 0.83) of the measuring instrument exceeded 0.83 [26]. The sampling frequency was 50 Hz. The subject stood still on the equipment with eyes open for 30 s 3 times. The subjects were then measured 3 times while standing forward for 30 s with closed eyes.

#### 2.4.2. Dynamic Balance Ability

The TUG test is used to evaluate the dynamic balance ability of stroke patients. The TUG test is a simple and rapid functional movement test, consisting of standing up, walking 3 m, returning, and sitting back down. It measures the time taken to sit in a chair with armrests and a backrest, stand up, walk 3 m, walk back, and return to sitting in the chair. This test measures the dynamic balance, functional movement, and gait ability of stroke patients with lower-extremity disabilities such as spasticity. It is a highly reliable and valid method for assessing risk (ICC = 0.99) [27]. In this experiment, a chair with a height of 50 cm was used for measurements. The measurements were performed three times using a stopwatch, and the mean value was calculated and recorded.

The FRT was used to evaluate stability limits. During this measurement, the subject stood approximately 10 cm away from the wall, bent their shoulder at 90°, made a fist, and extended their arm forward as far as possible parallel to the floor. The evaluation–revaluation reliability and inter-measurement reliability were high at r = 0.89 and r = 0.98, respectively [28]. In this experiment, three measurements were performed and the mean was recorded.

The BBS is used to measure balance in patients with stroke or older adults. The 14 items were scored on a 5-point scale from 0 to 4 for a total possible score of 56. This tool has high reliability and internal validity for assessing balance ability (r = 0.99 and r = 0.98, respectively) [29].

#### 2.4.3. Lower-Extremity Motor Function

Lower-extremity motor function was assessed in this study using the Fugl–Meyer assessment lower-extremity scale, which assesses functional recovery in patients with stroke. Each item on the test was rated on the following 3-point scale: 0 points for not being able to progress, 1 point for partial task completion, and 2 points for full task completion. The perfect score for lower-limb function was 34 points, and the test items consisted of the hip, knee, ankle, and coordination joints. Moreover, it is possible to evaluate balance, sensation, and pain; however, only lower-extremity function was considered in this study. This test has good inter- (r = 0.94) and intra-rater (r = 0.99) reliabilities [30].

#### 2.4.4. Activities of Daily Living (ADL)

The Modified Barthel Index (MBI) was used to evaluate the subjects’ ADL. The MBI is a scale that can be used to evaluate the level of performance in daily life. It includes the following 10 items: personal hygiene, bathing, feeding, toileting, stair climbing, dressing, bowel control, bladder control, ambulation, wheelchair transfer, and mobility. Each item was scored numerically according to the degree of assistance required by the individual. The maximum score for each figure is 100 points. A score of 0–24 indicates complete dependence, 25–49 indicates severe dependence, 50–74 indicates moderate dependence, and 75–90 indicates little dependence. The inter- (r = 0.99) and intra-rater (r = 0.99) reliabilities were high [31].

### 2.5. Data Analysis

The Statistical Package for the Social Sciences (SPSS version 19; IBM, Armonk, NY, USA) was used for the statistical analysis. To confirm the assumption of data normality, the Shapiro–Wilk test was used, which confirmed that the assumption of normality was satisfied. Two-way repeated measure ANOVA was used to analyze the main effects of “training type” (BT-ESG or BTG) and “time” on static and dynamic balance and function, and the interaction of treatment type and time was analyzed. Before and after comparisons within groups were analyzed using a paired t-test. Effect sizes were calculated to assess the strength of the training effect, and 95% confidence intervals, which reflect the actual changes in addition to errors, were calculated. The statistical significance level (α) was set at *p* < 0.05.

## 3. Results

### 3.1. General Characteristics of the Subjects

Sixty subjects were enrolled in this study. Of them, one from the BT-ESG dropped out, while all the subjects from the BTG completed the study. A total of 59 subjects completed the study. Before the intervention, the general characteristics of the two groups were homogeneous (Table 1).

### 3.2. Changes in Static Balance Ability

Changes in static balance ability, medial and lateral sway speed, anterior and posterior sway speed, and velocity of moment variables showed significant improvement after the intervention in both groups, regardless of the eyes-open or eyes-closed state (*p* < 0.05). However, the BT-ESG showed greater improvement than the BTG (*p* < 0.05) (Table 2).

### 3.3. Changes in Dynamic Balance Ability

The BT-ESG and BTG showed significant improvements in TUG and BBS scores after training, with significantly better improvements in the former versus latter (*p* < 0.05) (Table 3).

### 3.4. Changes in Lower-Extremity Motor Function and ADL

Both the BT-ESG and BTG showed significant improvements in the Fugl–Meyer assessment lower-extremity scale and MBI scores after training, with significantly better improvements in the former versus latter (*p* < 0.05) (Table 4).

## 4. Discussion

Electrical stimulation is a harmless method used to enhance the activation of residual muscles. In a study of stroke patients, electrical stimulation was used to recover patients with reduced function or paralysis [9,10]. In this study, it was applied to the four lower-extremity muscles that are necessary for weight support by triggering a weight shift, and muscle contraction was induced at an appropriate time to support weight support. Electrical stimulation induces direct muscle mobilization, unlike visual or auditory feedback [32] used in previous balance training studies. In this study, both groups showed significant improvements in static balance, dynamic balance, lower-extremity motor function, and ADL, and the BT-ESG showed significant improvement versus the BTG.

Maintaining postural balance is a complex task that requires appropriate sensory inputs and motor control [33]. An asymmetrical weight load causes the center of gravity to become unstable, increasing movement variability and making balancing difficult, owing to postural fluctuations. In this study, static balance ability was evaluated using postural sway, while dynamic balance ability was assessed using the TUG, FRT, and BBS. Both groups showed significant improvement, while the BT-ESG showed greater improvement than the BTG.

In this study, postural sway in the BT-ESG improved with a mediolateral speed increase of 28.2%, anterioposterior speed of 21.5%, velocity moment of 34.4% with eyes open and mediolateral speed of 26.2%, anterioposterior speed of 15.2% and velocity moment of 26.5% with eyes closed, while postural sway in the BTG improved with a mediolateral speed increase of 13.6%, anterioposterior speed of 10.9%, velocity moment of 17.8% with eyes open and a mediolateral speed of 11.6%, anterioposterior speed of 8.3% and velocity moment of 12.8% with eyes closed. In this study, postural sway in the BT-ESG showed more remarkable improvement compared to the BTG.

Even when proprioception is normal, postural sway increases more when the eyes are closed versus open [34]. In particular, when proprioception is impaired, such as in stroke patients, postural agitation becomes more severe when the eyes are closed [35]. Lobo et al. [36] showed that weight shift training and proprioceptive training improved the balance ability of stroke patients. Weight-bearing training, which induces a shift in the center of gravity toward the midline, reportedly improves joint stability, postural control, and balance by promoting load receptor feedback to the central nervous system and forcing the paralyzed limb to bear weight [36,37]. Cha et al. [38] reported that weight shift training is an intervention that can improve stroke patients’ proprioceptive and balance abilities. Moreover, applying somatosensory stimulation, such as electrical stimulation and manipulation of the surface beneath the feet to alter the proprioceptive input to the legs, effectively improves the sensory retraining and balance ability of stroke patients [8]. In this study, postural agitation improved more significantly in the BT-ESG than the BTG with electrical stimulation along with balance training, in which an insole-type pressure-measuring device was inserted under the foot of a chronic stroke patient and weight support was induced.

With dynamic balance, the TUG time showed a decrease of 14%. Since the TUG test includes gait, sitting, standing, and turning, balance ability is a predictor of gait [39]. The FRT showed an improvement of 23.6%, confirming improved stability. The BBS showed a 21.5% improvement, confirming that it is an intervention method that can mitigate the risk of falling. In this study, the progress of balance ability is thought to include improved postural symmetry by enhancing lower-limb muscle activation on the affected side and the weight-bearing capacity of the affected side. Previous studies reported that improving muscle tone, joint flexibility, and lower-extremity symmetry on the affected side improved balance ability [40,41,42].

Balance training with weight shift-triggered electrical stimulation was performed in stroke patients with asymmetrical weight-bearing, that is, asymmetrical plantar pressure, improved motor function, stimulated sensory input on the paralyzed leg, and repetitive use of the non-paretic leg. In this balance training program, weight shift-triggered electrical stimulation was provided so that the weight load could be symmetrically induced, while the center of the body moved in various directions in a standing position. Feedback made the subject aware of the incorrect posture, and their balance ability was improved because muscle contraction of the paretic leg was induced.

Alhirsan et al. [43] and Lin et al. [44] reported that repetitive task performance without feedback in stroke patients could continuously increase compensatory actions, create an inappropriate posture, and negatively restore motor function. In this study, the movement of hip extension on the affected side led to the improvement of dynamic balance by promoting weight-bearing and electrical stimulation on the affected side. When severe postural fluctuations occur while maintaining balance, the hip joint strategy is used, and the FRT evaluates the limit of stability while using the hip joint strategy as much as possible [28,45]. Balance training with weight shift-triggered electrical stimulation improved the dynamic balance ability by increasing the weight-bearing rate on the paralyzed side. Moreover, these results were consistent with those of a previous study that reported that dynamic balance was improved through weight shift training for stroke patients and a previous study reported that it was more beneficial to improve balance when combined with electrical stimulation balance training [46]. This finding is consistent with the results reported by Mahmoudi et al. [47].

A significant cause of postural balance problems in stroke patients is an imbalance in lower-extremity function and muscle activity between the affected and unaffected sides. The current study attempted to improve the lower-extremity function of the affected leg through balance training, which induces muscle contraction by triggering electrical stimulation of the affected leg. Since the Fugl–Meyer assessment lower-extremity scale evaluates voluntary movement and active contraction of the leg, it proved the effectiveness of this training for inducing muscle contractions in the affected leg [48]. In this study, the Fugl–Meyer assessment lower-extremity scale showed an improvement of 31.4%, confirming that the lower-extremity function was improved. Previous studies also reported that neuromuscular electrical stimulation and weight shift training reduced muscle tone through muscle activity on the affected side and improved lower-limb functional recovery [49,50]. Balance training with weight shift-triggered electrical stimulation is thought to lead to changes in lower-limb function by promoting the use of the affected side.

As for ADL, both groups showed significant improvement in the MBI score, but a greater improvement was observed in the BT-ESG. In previous studies, balance training seemed to have a positive effect by improving ADL in stroke patients [51]. These results are consistent with previous research results, showing that balance has a high correlation with ADL [52]. In this study, improved weight transfer ability improved balance ability and lower-limb function, which impacted daily life.

In this study, balance training with weight shift-triggered electrical stimulation improved balance ability and lower-extremity function, improving daily life movements. However, since no gait measurement was performed, it is difficult to conclude whether improvements in balance ability, lower-extremity function, and ADL are correlated with gait ability and the risk of falling. In addition, since all the study participants were chronic stroke patients with stroke onset of more than 1 year, the effect of the intervention cannot be generalized to all patients. Further research is needed to address these points.

## 5. Conclusions

Balance training with weight shift-triggered electrical stimulation, compared with balance training alone, resulted in significant improvements in static balance, dynamic balance, lower-extremity motor function, and ADL in chronic stroke patients. Since the primary outcome of this study used several primary variables for dynamic and static balance, multiplicity adjustment was clearly important. Our findings suggest that balance training with weight shift-triggered electrical stimulation can help to improve the postural symmetry and functional movements of stroke patients.

## Figures and Tables

**Table 1:** General characteristics of the subjects.

	BT-ESG (*n* = 29)	BTG (*n* = 30)	χ^2^/*t*	*p*
Age (year)	66.24 ± 7.01	68.87 ± 7.30	1.409	0.164
Height (cm)	163.48 ± 7.46	161.93 ± 10.20	0.664	0.509
Weight (kg)	60.50 ± 8.01	60.93 ± 8.64	0.199	0.843
Body mass index (point)	22.60 ± 2.34	23.16 ± 1.81	1.033	0.306
Duration of stroke (month)	14.17 ± 5.87	16.33 ± 5.84	1.417	0.162
MMSE	25.83 ± 1.23	25.53 ± 1.01	1.008	0.318
MBI	52.99 ± 8.52	54.92 ± 8.96	0.846	0.401
Gender (male/female)	16/13	17/13	0.013	0.908
Paretic side (right/left)	15/14	20/10	0.243	1.364
Stroke type (infarction/hemorrhage)	19/10	18/12	0.661	0.192

Values are expressed as mean ± standard deviation. The independent *t*-test and chi-squared tests were used to compare the dependent variables between the two groups. BT-ESG, balance training with electrical stimulation group; BTG, balance training group; MMSE, Mini-Mental State Examination; MBI, Modified Barthel Index.

**Table 2:** Changes in static balance ability.

		BT-ESG (*n* = 29)	BTG (*n* = 30)	Time *F*(*p*)	Interaction *F*(*p*)	Effect Size (d)	CI for Differences	
							Lower	Upper
EO M-L speed (mm/s)	Pre	4.08 ± 1.36	4.12 ± 1.36					
	Post	2.93 ± 0.66	3.56 ± 1.02	50.691	6.115		−1.07	−0.11
	Pre–Post	−1.15 ± 1.01 *	−0.56 ± 0.83 *	(0.000)	(0.016)	0.64		
EO A-P speed (mm/s)	Pre	5.99 ± 1.49	6.17 ± 1.28					
	Post	4.70 ± 1.42	5.50 ± 1.33	127.546	12.789		−0.97	−0.27
	Pre–Post	−1.30 ± 0.76 *	−0.67 ± 0.57 *	(0.000)	(0.001)	0.93		
EO Velocity moment (mm^2^/s)	Pre	4.33 ± 1.41	4.28 ± 1.17					
	Post	2.84 ± 1.40	3.52 ± 1.24	108.358	11.436		−1.16	−0.30
	Pre–Post	−1.48 ± 0.69 *	−0.76 ± 0.94 *	(0.000)	(0.001)	0.88		
EC M-L speed (mm/s)	Pre	4.35 ± 1.36	4.39 ± 0.97					
	Post	3.21 ± 0.93	3.88 ± 1.11	83.943	12.052		−0.99	−0.26
	Pre–Post	−1.14 ± 0.75 *	−0.51 ± 0.63 *	(0.000)	(0.001)	0.90		
EC A-P speed (mm/s)	Pre	5.92 ± 1.76	5.57 ± 0.81					
	Post	5.02 ± 1.42	5.11 ± 0.91	91.159	9.877		−0.73	−0.16
	Pre–Post	−0.09 ± 0.57 *	−0.46 ± 0.53 *	(0.000)	(0.003)	0.82		
EC Velocity moment (mm^2^/s)	Pre	5.02 ± 1.96	4.61 ± 1.79					
	Post	3.69 ± 1.47	4.02 ± 1.68	64.438	9.766		−1.22	−0.27
	Pre–Post	−1.33 ± 0.94 *	−0.58 ± 0.88 *	(0.000)	(0.003)	0.81		

Values are expressed as mean ± standard deviation. * means significant difference within groups. BT-ESG, balance training with electrical stimulation group; BTG, balance training group; EO, eyes open; EC, eyes closed; M-L, mediolateral; A-P, anterioposterior; CI, confidence interval.

**Table 3:** Changes in dynamic balance ability.

		BT-ESG (*n* = 29)	BTG (*n* = 30)	Time *F*(*p*)	Interaction *F*(*p*)	Effect Size (d)	CI for Differences	
							Lower	Upper
TUG (sec)	Pre	36.05 ± 4.41	34.58 ± 4.58					
	Post	30.99 ± 3.99	32.48 ± 5.11	0.552	12.014		−4.67	−1.25
	Pre–Post	−5.06 ± 2.74 *	−2.10 ± 3.72 *	(0.000)	(0.000)	0.90		
BBS (point)	Pre	28.69 ± 6.89	29.70 ± 7.37					
	Post	34.86 ± 6.45	31.03 ± 6.75	56.721	23.577		2.84	6.83
	Pre–Post	6.17 ± 4.71 *	1.33 ± 2.71 *	(0.000)	(0.000)	1.26		
FRT (cm)	Pre	14.56 ± 3.73	12.89 ± 3.53					
	Post	17.99 ± 3.82	13.32 ± 3.84	51.188	30.922		1.92	4.08
	Pre–Post	3.43 ± 2.72 *	0.43 ± 1.13 *	(0.000)	(0.022)	1.45		

Values are expressed as mean ± standard deviation. * means significant difference within groups. BT-ESG, balance training with electrical stimulation group; BTG, balance training group; TUG, Timed Up and Go test; BBS, Berg Balance Scale; FRT, functional reach test; CI, confidence interval.

**Table 4:** Changes in lower-extremity motor function and ADL.

		BT-ESG (*n* = 29)	BTG (*n* = 30)	Time *F*(*p*)	Interaction *F*(*p*)	Effect Size (d)	CI for Differences	
							Lower	Upper
FMA (point)	Pre	15.10 ± 3.03	14.74 ± 3.22					
	Post	19.84 ± 4.57	15.87 ± 2.64	74.366	28.130		2.25	4.97
	Pre–Post	4.74 ± 3.39 *	1.13 ± 1.53 *	(0.000)	(0.000)	1.38		
MBI (score)	Pre	52.99 ± 8.52	54.92 ± 8.96					
	Post	60.79 ± 12.58	57.01 ± 8.10	71.552	23.817		2.04	9.37
	Pre–Post	7.80 ± 5.72 *	2.09 ± 8.10 *	(0.000)	(0.000)	0.81		

Values are expressed as mean ± standard deviation. * means significant difference within groups. BT-ESG, balance training with electrical stimulation group; BTG, balance training group; FMA, Fugl–Meyer assessment; MBI, Modified Barthel Index; CI, confidence interval.
